# No evidence for general or food-specific inhibitory control deficits in overweight university students: findings from flanker and Food Go/Nogo tasks

**DOI:** 10.3389/fpsyg.2026.1781483

**Published:** 2026-03-17

**Authors:** Ningqi Yang, Hang Zheng, Bo Sun

**Affiliations:** 1 School of Physical Education, Guangxi University, Nanning, Guangxi, China; 2 School of Athletic Performance, Shanghai University of Sport, Shanghai, China

**Keywords:** food cues, inhibitory control, overweight, response inhibition, university students

## Abstract

**Introduction:**

The transition from normal weight to obesity, defined as being “overweight,” represents a critical window for preventive intervention; however, it remains debated whether cognitive vulnerabilities at this stage manifest as a domain-general decline in executive function or a domain-specific hypersensitivity to food cues.

**Methods:**

To address this, the present study employed a cross-sectional design to systematically investigate the behavioral profiles of general and food-specific inhibitory control in overweight university students. Twenty-eight participants (14 overweight and 14 normal-weight) completed the classic Flanker task and a modified Food Go/Nogo task to assess interference control and response inhibition, respectively.

**Results:**

The results revealed no significant differences between the groups in reaction time or accuracy across both tasks, indicating that inhibitory function remains behaviorally intact in overweight individuals. Notably, both groups demonstrated significantly higher inhibition accuracy for high-calorie food images compared to low-calorie ones, suggesting the operation of a “defensive inhibition” mechanism rather than a reward-driven control failure.

**Discussion:**

These findings support a dose-dependent model of neuropathological impact, implying that the “overweight” stage serves as a prodromal phase where cognitive control is preserved—potentially bolstered by the cognitive reserve characteristic of this educated cohort—thus identifying a crucial opportunity for early lifestyle interventions before the onset of significant impairment.

## 1 Introduction

The escalating prevalence of overweight and obesity presents a critical global public health challenge (Blüher, 2019). Beyond its well-established risks for cardiovascular and metabolic disorders, excessive body weight is inextricably linked to cognitive alterations, most notably in inhibitory control. Defined as the core executive function enabling the regulation of impulses and the suppression of prepotent responses, inhibitory control is decisive for successful weight management (Fitzpatrick et al., 2013). Neurocognitive models posit that the prefrontal cortex (PFC) exerts top-down regulation over appetite; compromised PFC function may lead to a failure in suppressing bottom-up reward responses to high-calorie foods, thereby facilitating overeating (Lowe et al., 2020). Furthermore, chronic energy surplus can structurally and functionally impair the brain, fueling a bidirectional vicious cycle between cognitive decline and weight gain (Volkow et al., 2011; Higgs and Spetter, 2018).

Although the cognitive disparities between healthy weight and clinically obese individuals are well-documented, there is a distinct scarcity of research focusing on the “overweight” category—a pivotal transitional stage. Defined as a BMI of 24–28 kg/m^2^ (Jiang et al., 2025), being overweight represents a critical intervention window before the onset of obesity. National surveillance data in China indicate a marked increase in overweight and obesity among college students. Based on national cross-sectional surveys (CNSSCH) from 1995 to 2019, the prevalence of overweight rose from 3.7 to 23.4%, and obesity increased from 0.7 to 11.4% among Chinese college students aged 19–22 years, underscoring this population as a relevant group for investigating cognitive-behavioral factors related to elevated body weight (Du et al., 2026). This transition is particularly pertinent to university students, a demographic undergoing a sensitive period of lifestyle consolidation and neurocognitive maturation (Deforche et al., 2015; Winpenny et al., 2020). Faced with academic stressors and the demands of independent living, students are uniquely vulnerable to obesogenic environments. Excess weight during this phase serves not only as a precursor to long-term metabolic risks but may also associate with subtle deficits in inhibitory function, potentially undermining academic achievement and social adaptation (Li et al., 2008; Lowe et al., 2020). Unlike clinical obesity, which is often characterized by established pathology, the cognitive impairments in overweight individuals are likely prodromal or subclinical (Yang et al., 2018). Consequently, characterizing inhibitory profiles in overweight university students is of paramount importance for preventive strategies aiming to arrest the trajectory toward obesity and protect cognitive and academic well-being.

Inhibitory control is not a unitary construct but rather a multidimensional executive function comprising two distinct components: interference control (the ability to suppress cognitive interference from distractors, e.g., the Flanker task) and response inhibition (the ability to cancel or withhold a prepotent motor response, e.g., the Go/Nogo task) (Diamond, 2013). A central controversy in current obesity research concerns the specificity of inhibitory deficits in overweight individuals (Smith et al., 2011). The “general deficit” perspective posits that excess body weight is linked to a generalized impairment in executive functioning, resulting in domain-general inhibitory deficits. In contrast, the “reward-specific” perspective argues that cognitive control remains intact globally but is selectively compromised by high-calorie food cues—a phenomenon attributed to the heightened incentive salience of food stimuli (Finlayson et al., 2007; Price et al., 2016).

Consequently, the prevailing ambiguity regarding the nature of inhibitory dysfunction—whether it represents a domain-general impairment or a food-specific vulnerability—hampers the development of targeted therapeutic strategies (Vainik et al., 2013). A domain-general deficit would necessitate interventions aimed at bolstering the global prefrontal executive control network (Allom et al., 2016). In contrast, a domain-specific deficit, likely stemming from heightened incentive salience of food cues, would require protocols focused on devaluation strategies or food-specific inhibitory control training (Lawrence et al., 2015; Veling et al., 2017). Clarifying this dichotomy is therefore essential for elucidating the neurocognitive etiology of overweight and informing precision weight management interventions (Ek et al., 2023).

Based on this rationale, the present study employs a cross-sectional design aimed at systematically investigating the behavioral characteristics of inhibitory function in overweight university students. By combining the classic Flanker task with a modified Food Go/Nogo task to assess domain-general interference control and food-specific response inhibition respectively, this study seeks not only to clarify whether specific cognitive control deficits exist at the overweight stage but also to provide necessary theoretical evidence and reference for selecting matched intervention modalities (e.g., targeted exercise prescriptions or cognitive training) based on distinct cognitive profiles in future research. Following hypothesis-driven methodology, we formulated *a priori*, falsifiable predictions: if inhibitory dysfunction at the overweight stage is domain-general, overweight participants should exhibit a larger Flanker interference cost; if vulnerability is food-cue specific, deficits should manifest primarily as reduced Nogo accuracy for high-calorie food cues. Alternatively, salient high-calorie cues may elicit counter-regulatory top-down control, resulting in preserved or enhanced inhibitory performance in high-calorie versus low-calorie conditions.

## 2 Materials and methods

### 2.1 Participants

Participants were recruited via social media platforms (e.g., QQ groups), campus posters, and student organizations To minimize potential confounds, the inclusion criteria required participants to have: (1) normal or corrected-to-normal vision; and (2) no self-reported history of psychiatric or neurological disorders (including ADHD and brain injury), no history of clinically diagnosed eating disorders, or chronic metabolic/cardiovascular conditions. In addition, participants reported no current mental health conditions and no use of psychoactive substances that could influence cognitive performance (e.g., caffeine, nicotine) on the day of testing. Exclusion criteria included the use of medications affecting metabolism or weight within the past 6 months. Additionally, participants were screened for physical activity levels to ensure homogeneity. Physical activity was assessed using the two-item Physical Activity Vital Sign/Exercise Vital Sign (PAVS/EVS), which records (1) the number of days per week and (2) the average minutes per day of moderate-to-vigorous physical activity (MVPA) during an average week in the past 30 days; weekly MVPA (min/week) was calculated as days × min (Coleman et al., 2012; Kuntz et al., 2021). Based on the World Health Organization (WHO) recommendations, activity levels were categorized as inactive (<150 min/week), moderately active (150–299 min/week), or highly active (≥300 min/week) (Bull et al., 2020). The participant-level screening data are provided in Supplementary material. Group classification was based on Body Mass Index (BMI), with the overweight group defined as 24 kg/m^2^ (Pan et al., 2021).

Sample size was determined via an *a priori* power analysis using G*Power 3.1 (Faul et al., 2007) to determine the requisite sample size. Based on the 2 (Group) × 3 (Image Type) mixed factorial design, the parameters were set as follows: a medium effect size (*f* = 0.25), a significance level (*α* = 0.05), and a statistical power (1−*β* = 0.80). The analysis indicated that a minimum total sample size of 28 participants was required to detect significant interaction effects. Consequently, the final sample of 28 participants (14 per group) was deemed sufficient for statistical validity.

A total of 33 individuals were initially screened, of whom 5 were excluded for failing to meet the inclusion criteria. Consequently, 28 participants were enrolled in Experiment 1, consisting of 14 in the normal weight group and 14 in the overweight group. The final sample included 14 males and 14 females, with an equal sex distribution in each group (NW: 7 males, 7 females; OW: 7 males, 7 females). All participants received financial compensation upon completion of the experiment. The study was approved by the Ethics Committee of Central China Normal University (Approval No. CCNU-IRB-20230627). Participants were classified into two groups: the normal weight group (NW) and the overweight group (OW). These abbreviations are used consistently throughout the text.

### 2.2 Experimental design

The present study employed a cross-sectional design to assess baseline differences in inhibitory control between normal-weight and overweight university students. No exercise or dietary interventions were administered in this phase. The study consisted of two distinct task paradigms:

General Inhibitory Control Task: 2 (Group: NW, OW) × 2 (Congruency: Congruent, Incongruent) mixed factorial design was utilized. Group served as the between-subjects variable based on the participants’ natural BMI status, while Congruency served as the within-subjects variable. The dependent variables were reaction time (RT) and response accuracy (ACC) derived from the Flanker task.

**Figure 1:**
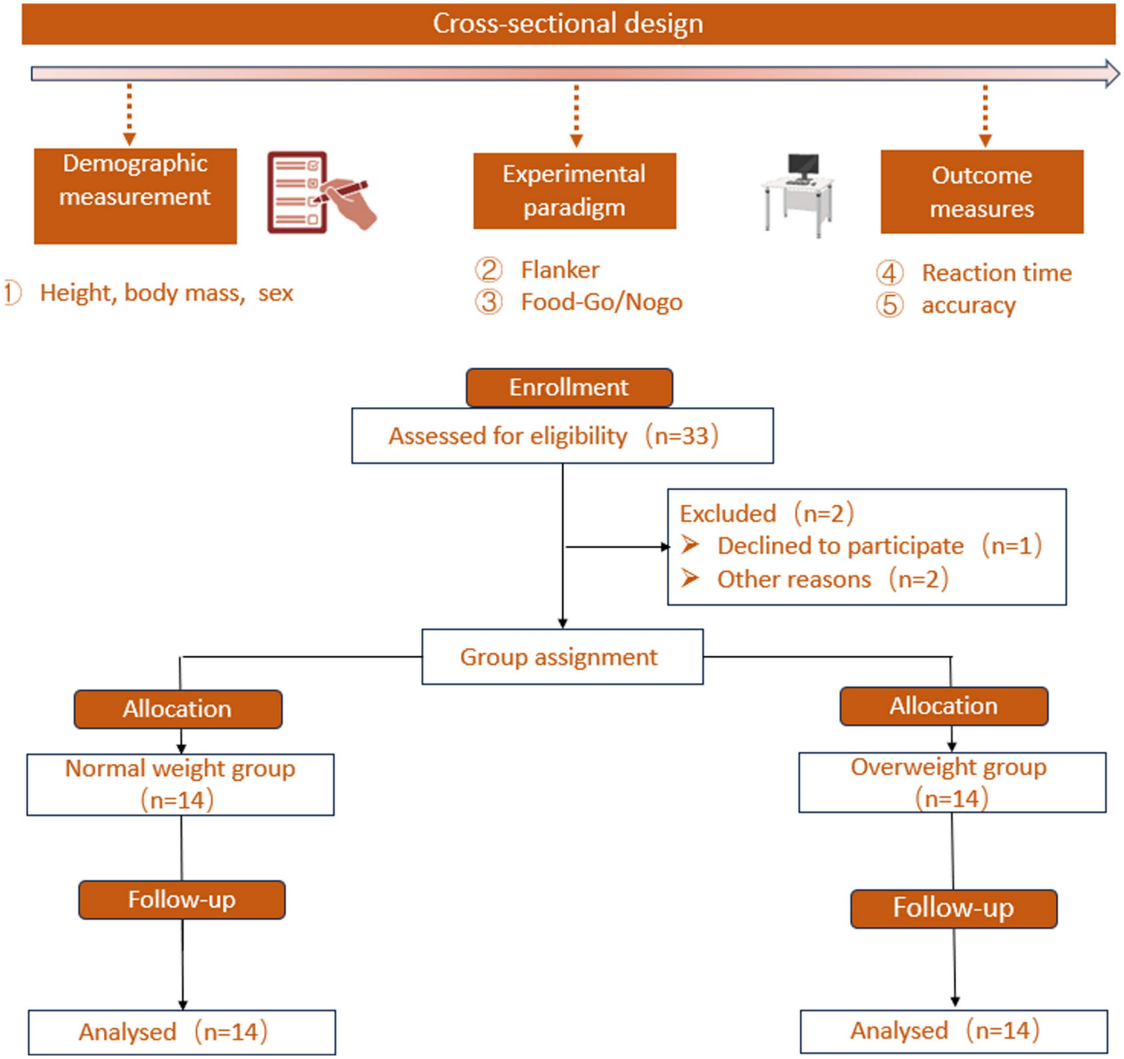
Flowchart of experimental design and participant recruitment.

Food-specific inhibitory control task: 2 (Group: NW, OW) × 3 (Image Type: High-Calorie, Low-Calorie, Neutral) mixed factorial design was employed. Group served as the between-subjects variable, and Image Type served as the within-subjects variable. The dependent variables included reaction time for Go trials (Go RT), accuracy for Go trials (Go ACC), and accuracy for Nogo trials (Nogo ACC) derived from the Food Go/Nogo task, as shown in Figure 1.

### 2.3 Stimuli and apparatus

The experimental tasks were programmed and presented using E-Prime 3.0 software (Psychology Software Tools, Pittsburgh, PA), which was also used to record behavioral data (RT and ACC) for both the Flanker and Food Go/Nogo tasks. All tests were administered on an HP desktop computer.

### 2.4 Experimental paradigms

**Figure 2:**
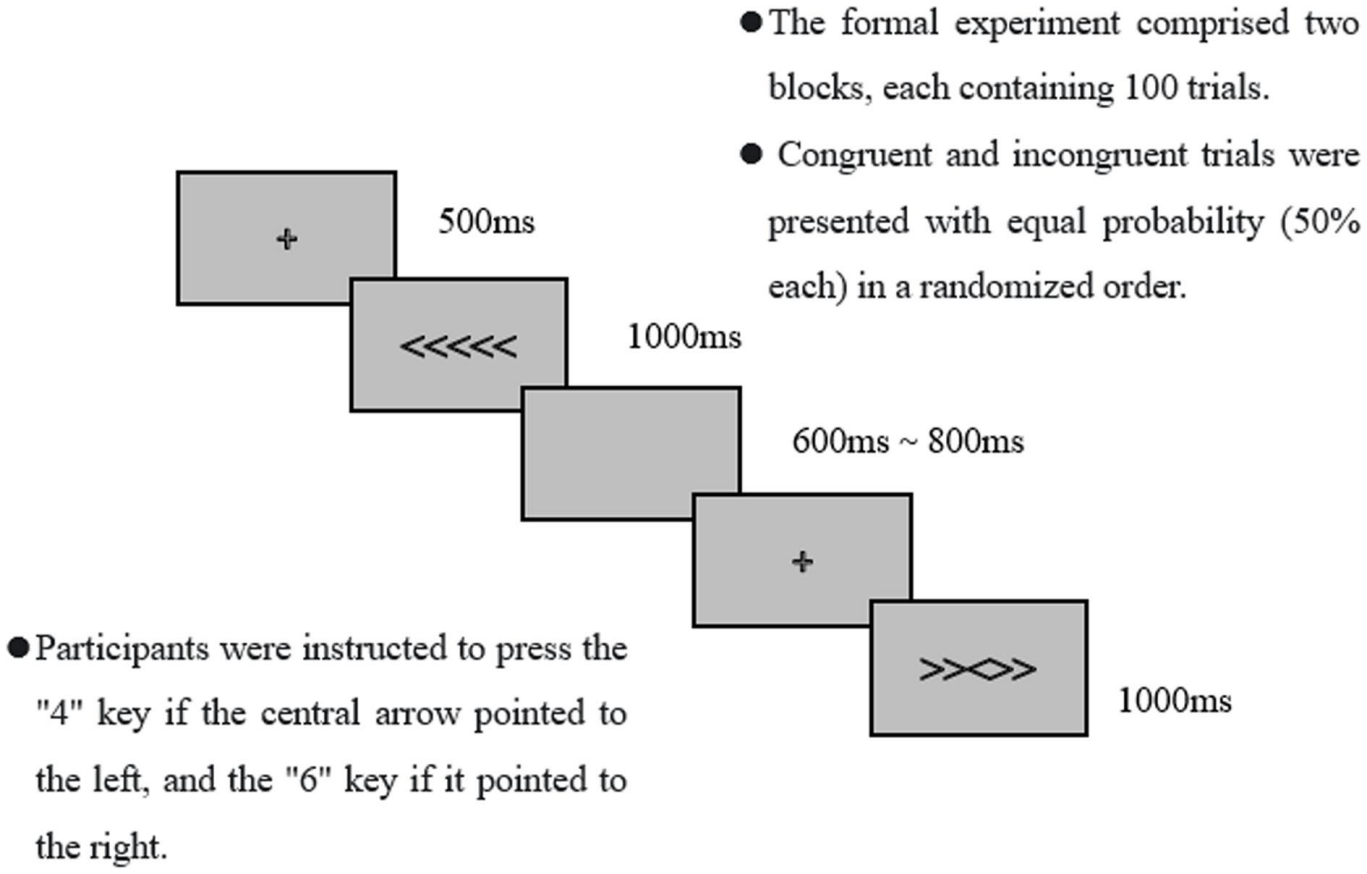
Schematic representation of the Flanker task.

The Flanker task: a modified version of the Eriksen Flanker task was adopted (Eriksen and Eriksen, 1974). The stimuli were presented on a white background. A fixation cross (+) appeared first to focus the participants’ attention, followed by the target stimulus. The stimulus consisted of a horizontal row of five arrows, and participants were required to identify the direction of the central arrow. Two stimulus conditions were included: Congruent (e.g., <<<<< or >>>>>) and Incongruent (e.g., <<><< or >><>>). Participants placed their right index finger on the “5” key of the numeric keypad. They were instructed to press the “4″ key if the central arrow pointed left and the “6” key if it pointed right. After each response, participants returned their finger to the “5” key. A practice session was conducted under the supervision of the experimenter prior to the formal test to ensure familiarity with the procedure, as shown in Figure 2.

**Figure 3:**
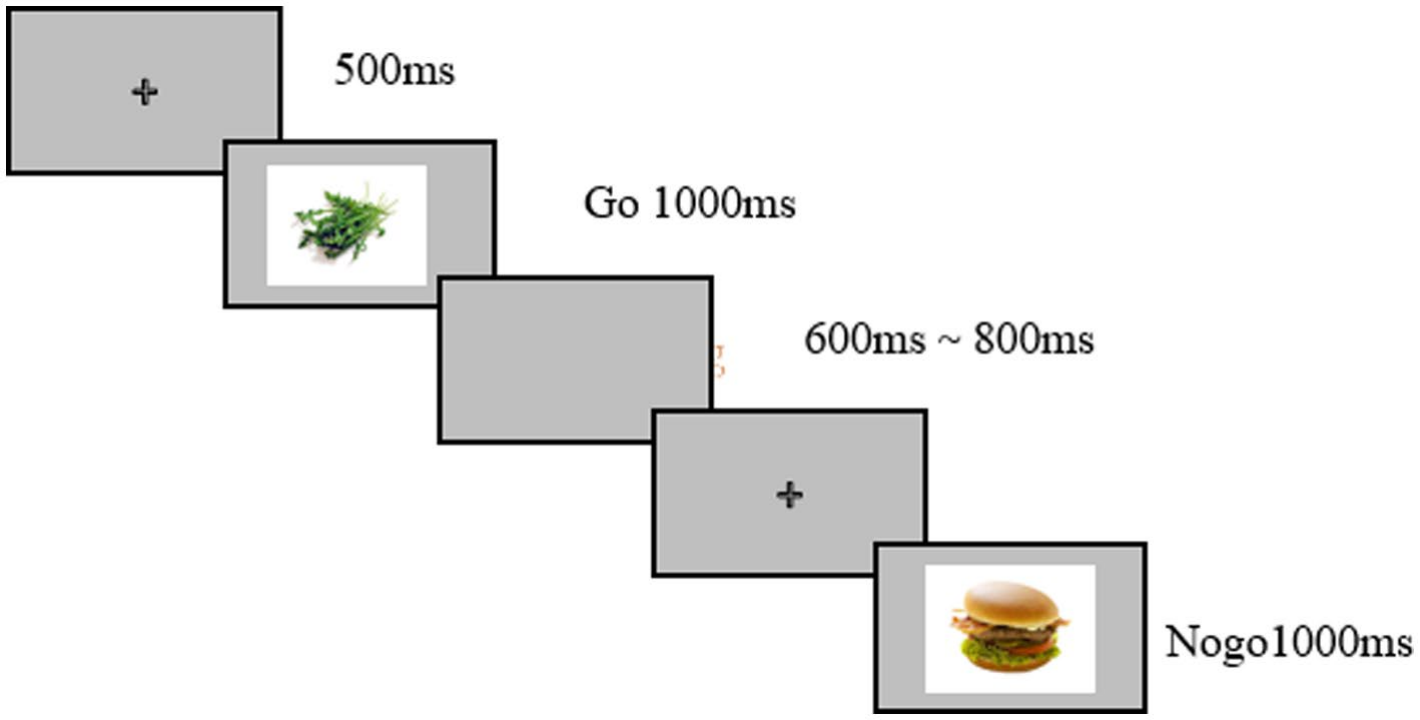
Schematic representation of the Food Go/Nogo task.

The Food Go/Nogo task: a modified Food Go/Nogo paradigm was utilized (Blechert et al., 2014; Carbine et al., 2017). Stimuli were selected from the standardized food picture database developed by Blechert et al. (2014). A total of 180 images were selected, comprising 60 high-calorie food images, 60 low-calorie food images, and 60 neutral images. The task consisted of six blocks, presented in a random order, with different combinations of stimulus categories as Go or Nogo targets: (1) Go: high-calorie/Nogo: neutral; (2) Go: Neutral/Nogo: high-calorie; (3) Go: high-calorie/Nogo: low-calorie; (4) Go: low-calorie/Nogo: high-calorie; (5) Go: low-calorie/Nogo: neutral; and (6) Go: Neutral/Nogo: low-calorie. A practice block containing 10 trials preceded the six formal experimental blocks. Image types were presented randomly within blocks. The probability of Go trials was 75%, and Nogo trials was 25%, as shown in Figure 3.

### 2.5 Procedure

The experiment was conducted in a behavioral psychology laboratory. Prior to testing, participants provided written informed consent, and their demographic information (e.g., age, BMI) was recorded. Body height and weight were measured onsite in the laboratory on the same day as the behavioral testing. Height was assessed using a portable stadiometer (SECA 213; SECA GmbH & Co. KG, Hamburg, Germany) to the nearest 0.1 cm, with participants barefoot and standing upright in the Frankfurt plane. Body weight was measured using a calibrated digital scale (SECA 803; SECA GmbH & Co. KG, Hamburg, Germany) to the nearest 0.1 kg, with participants wearing light clothing and without shoes. For each measure, two readings were taken and averaged; if the two readings differed by more than 0.5 cm for height or 0.2 kg for weight, a third measurement was taken and the mean of the two closest values was used. Body mass index (BMI) was calculated as weight (kg) divided by height squared (m^2^). All measurements were obtained by trained research assistants following a standardized protocol, and the scale was calibrated at the start of each testing day. To control for order effects and fatigue, the presentation order of the Flanker task and the Food Go/Nogo task was counterbalanced across participants. Testing sessions were scheduled between 8:30–11:30 a.m. and 2:30–5:30 p.m. To minimize variability in hunger/satiety that could influence food-related task performance, participants were tested in a non-fasted but intake-standardized state. They were instructed to refrain from eating and from consuming caloric beverages for at least 2 h prior to the session (water permitted).

### 2.6 Data analysis

Demographic characteristics (age, height, weight, and BMI) of the NW and OW groups were collected. Descriptive statistics were calculated and reported as mean ± standard deviation (M ± SD). To confirm that the NW and OW groups were distinct on the key anthropometric indicators used for group classification, between-group differences in body weight and BMI were examined using independent-samples *t*-tests (Welch’s correction was applied when homogeneity of variance was violated). Hedges’ g was calculated to estimate the effect size for these between-group comparisons.

For behavioral data, reaction time (RT) and accuracy (ACC) for congruent and incongruent conditions in the Flanker task were extracted after outlier removal. For the Food Go/Nogo task, Go RT, Go ACC, and Nogo ACC were analyzed, behavioral outcomes are summarized as mean ± SD. Statistical analyses were performed using SPSS 20.0. For the Flanker task, a 2 (Group) × 2 (Congruency) repeated measures analysis of variance (ANOVA) was conducted. As a supplementary (secondary) categorical check, we reported the number and percentage of participants contributing valid data to the congruent and incongruent conditions in the Flanker task. We also constructed a 2 × 2 contingency table to examine whether overweight status was associated with a higher proportion of participants showing incongruent incorrect responses (defined as any error on incongruent trials; i.e., incongruent ACC < 1.00), and performed a chi-square test (with Fisher’s exact test when expected cell counts were small).

For the Food Go/Nogo task, separate 2 (Group) × 3 (Image Type) repeated measures ANOVAs were conducted for Go RT, Go ACC, and Nogo ACC. Mauchly’s test was used to assess the assumption of sphericity; if violated, the degrees of freedom and *p*-values were corrected using the Greenhouse–Geisser method. *Post-hoc* comparisons were performed using the Bonferroni correction. The significance level was set at 0.05, and partial eta squared was reported as the measure of effect size. Significance levels were denoted as follows: ^*^p < 0.05, ^**^p < 0.01, and ^***^p < 0.001.

## 3 Results

### 3.1 Demographic characteristics

Descriptive statistics were calculated for the age, height, weight, and Body Mass Index (BMI) of the normal weight (NW) and overweight (OW) groups. All participants were current university students, and their BMI values met the established criteria for their respective classifications. The final sample consisted of 14 participants in the NW group and 14 participants in the OW group. The demographic and anthropometric characteristics of the participants, including age, height, weight, and BMI, are presented in Table 1. To statistically verify group distinctiveness on the key anthropometric indicators, independent-samples *t*-tests (Welch’s correction when variances were unequal) were performed for body weight and BMI. The OW group showed significantly higher body weight than the NW group (*p* < 0.05) and a markedly higher BMI than the NW group (*p* < 0.001), Consistent with these tests, effect sizes indicated a moderate-to-large between-group difference in body weight (Hedges’ *g* = 0.77) and a large between-group difference in BMI (Hedges’ *g* = 1.55).

**Table 1:** Demographic and anthropometric characteristics of the participants (mean ± SD).

Variable	Total (*n* = 28)	Normal-weight group (NW, *n* = 14)	Overweight group (OW, *n* = 14)
Male	14	7	7
Female	14	7	7
Age (years)	22.75 ± 2.50	24.21 ± 1.19	21.28 ± 2.64
Height (cm)	170.57 ± 9.44	171.92 ± 9.84	169.21 ± 9.18
Weight (kg)	67.30 ± 14.83	61.75 ± 11.14	72.85 ± 16.32^*^
BMI (kg/m^2^)	22.96 ± 3.57	20.72 ± 1.94	25.19 ± 3.46^***^

Between-group differences were tested using independent-samples *t* tests (Welch). ^*^p < 0.05; ^***^p < 0.001 vs. NW.

### 3.2 General inhibitory function

#### 3.2.1 Reaction time

**Figure 4:**
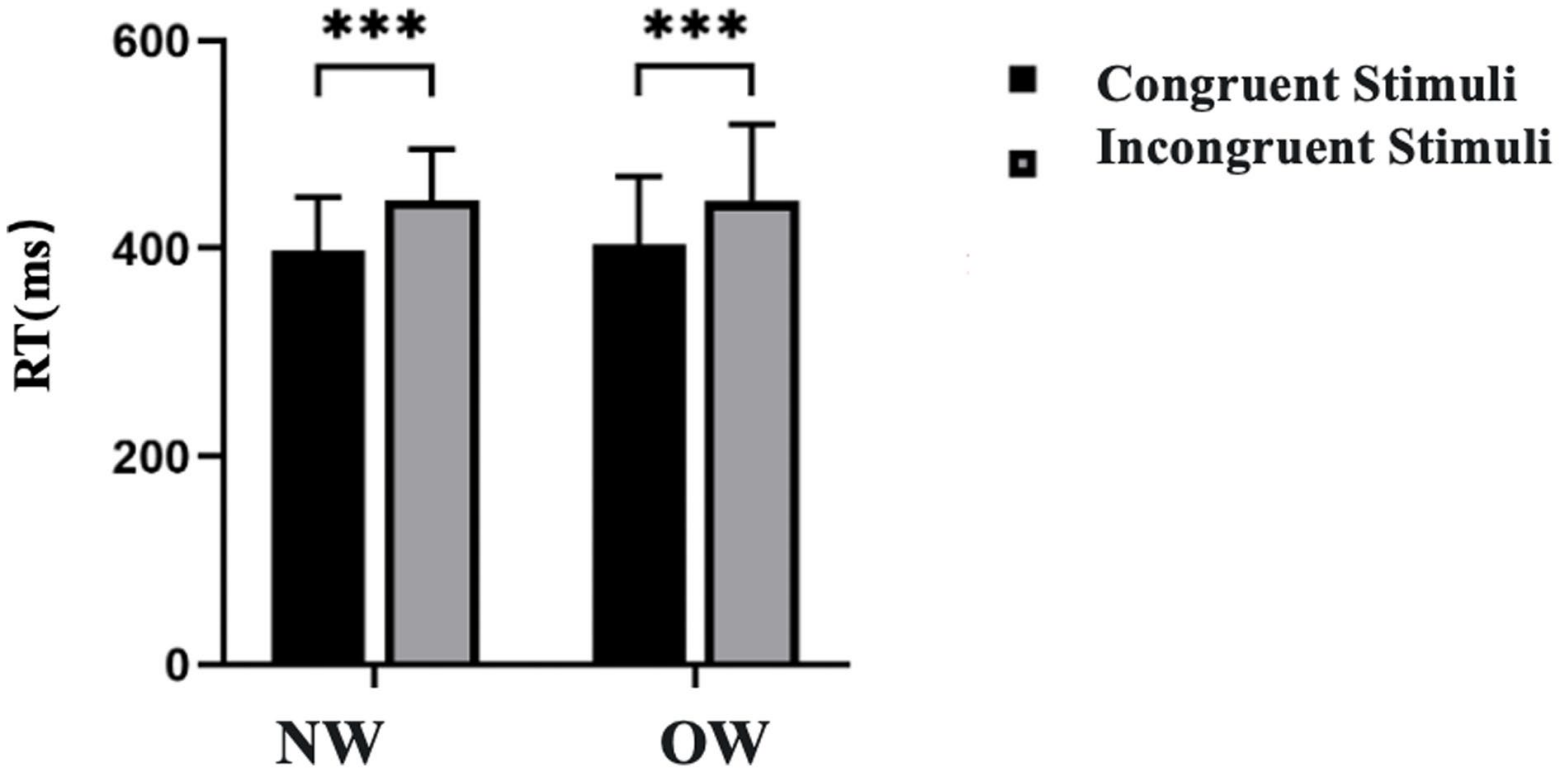
Reaction times for the Flanker task in normal weight and overweight university students. Data are expressed as mean ± SD. NW, normal-weight group; OW, overweight group; RT, reaction time. ^***^p < 0.001 for congruent vs. incongruent comparisons.

2 (Group: NW, OW) × 2 (Congruency: Congruent, Incongruent) repeated measures ANOVA was conducted on the reaction time (RT) and accuracy of the Flanker task:

Regarding Reaction Time, a significant main effect of Congruency was observed [*F*(1, 26) = 69.716, *p* < 0.001, *η^2^_P_* = 0.729]; Participants exhibited significantly slower reaction times in the incongruent condition compared to the congruent condition (446.571 ± 11.798 vs. 401.440 ± 11.098). However, the main effect of Group was not significant [*F*(1, 26) = 0.013, *p* = 0.910, *η^2^_P_* = 0.001]; furthermore, no significant interaction effect was found between Group and Congruency [*F*(1, 26) = 0.302, *p* = 0.587, *η^2^_P_* = 0.011], as shown in Figure 4.

#### 3.2.2 Response Accuracy

**Figure 5:**
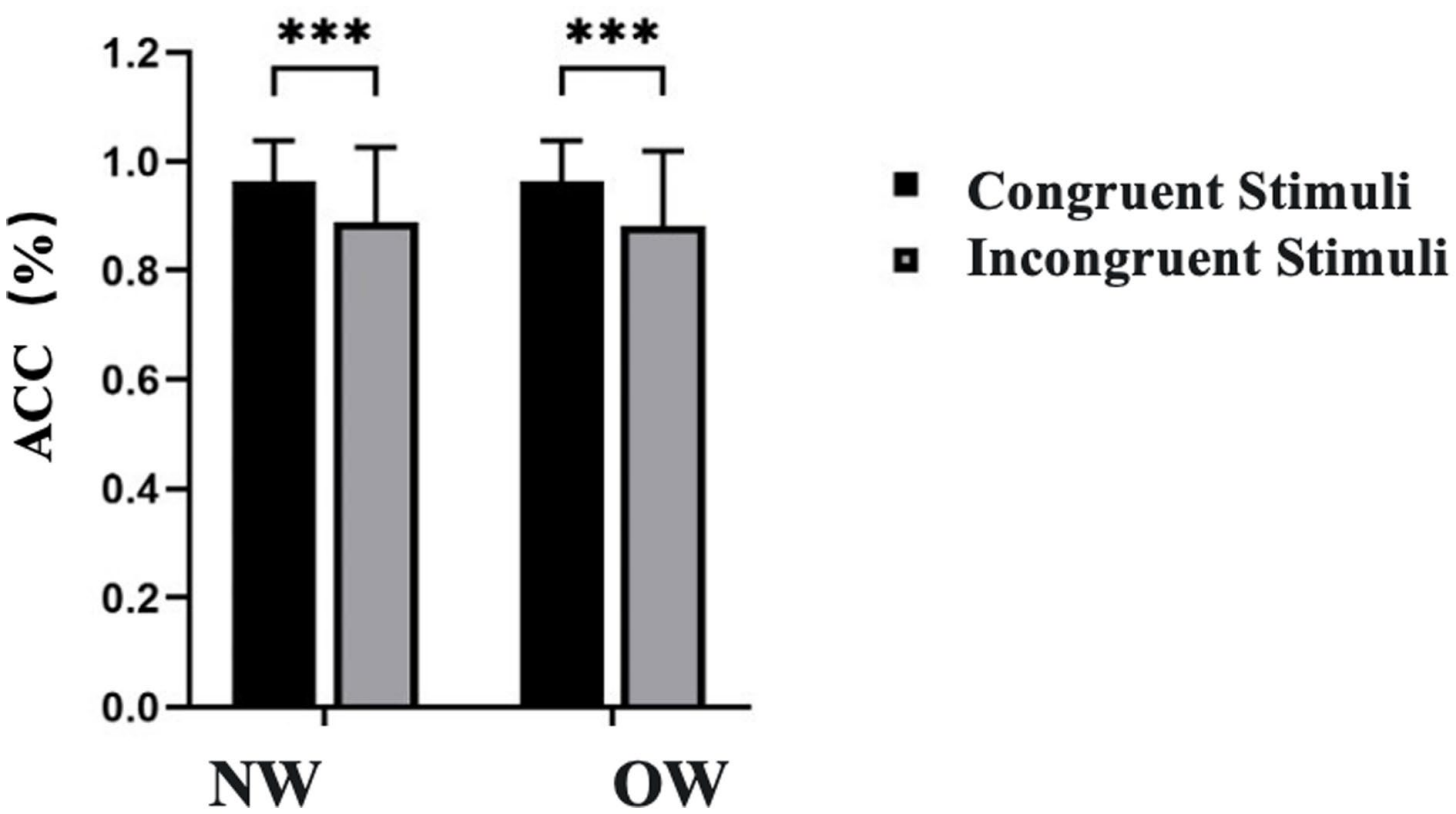
Reaction accuracy for the Flanker task in normal weight and overweight university students. Data are expressed as mean ± SD. NW, normal-weight group; OW, overweight group; ACC, accuracy. ^***^p < 0.001 for congruent vs. incongruent comparisons.

Regarding Response Accuracy, results indicated a significant main effect of Congruency [*F*(1, 26) = 24.187, *p* < 0.001,*η^2^_P_* = 0.502]. Specifically, accuracy was significantly higher in the congruent condition (0.965 ± 0.014) compared to the incongruent condition (0.886 ± 0.026). However, the main effect of Group was not significant [*F*(1, 26) = 0.007, *p* = 0.932, *η^2^_P_* < 0.001]. Additionally, the interaction between Group and Congruency was not significant (*p* > 0.05),as shown in Figure 5.

### 3.3 Food-specific inhibitory function

2 (Group: NW, OW) × 3 (Image Type: high-calorie, low-calorie, neutral) repeated measures ANOVA was conducted on Go Reaction Time, Go Accuracy, and Nogo Accuracy derived from the Food Go/Nogo task.

#### 3.3.1 Go Reaction Time

**Figure 6:**
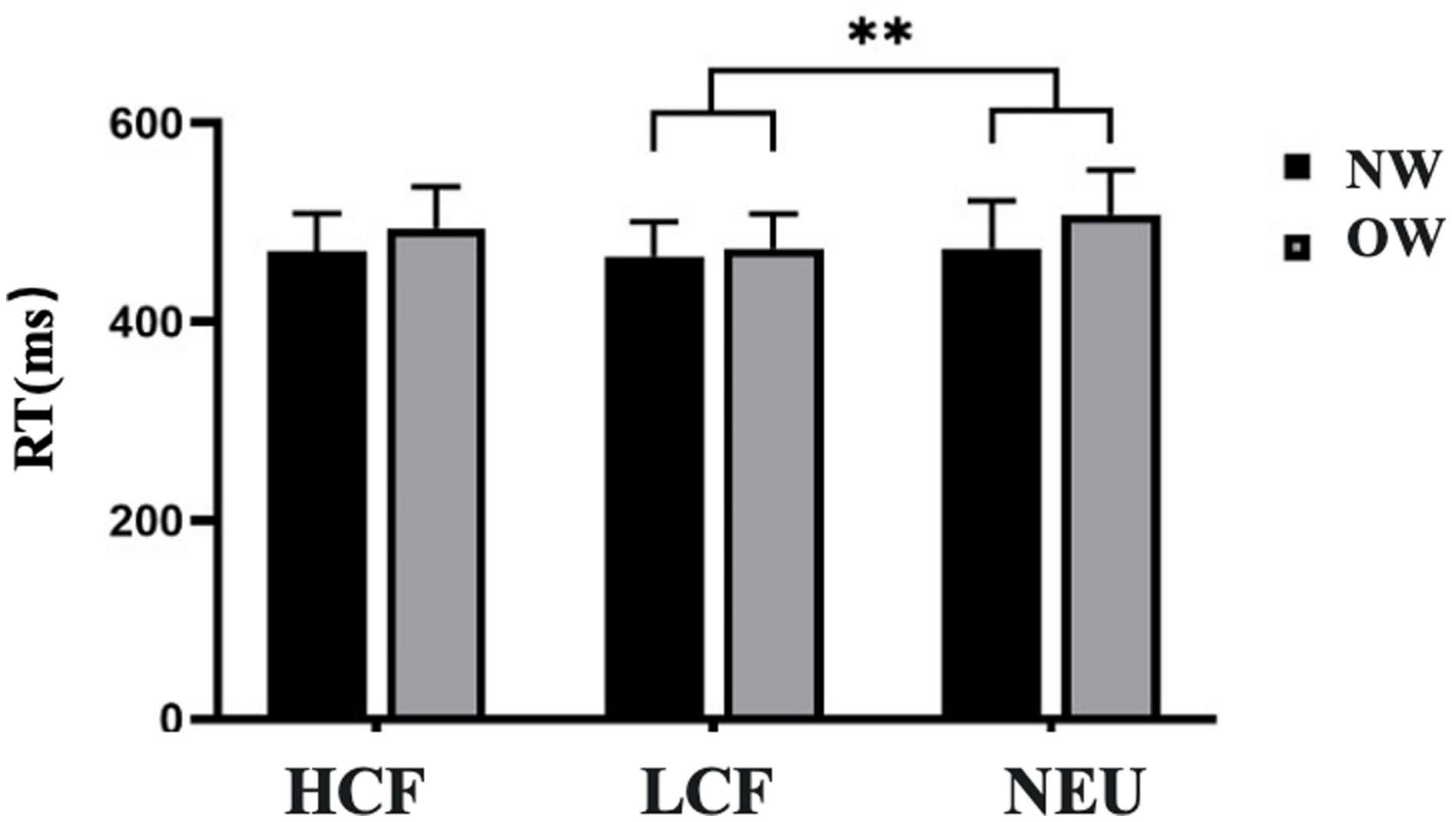
Go Reaction Times for the Food Go/Nogo task in normal weight and overweight university students. Task conditions comprised high-calorie food (HCF), low-calorie food (LCF), and neutral images (NEU). Data are expressed as mean ± SD. NW, normal-weight group; OW, overweight group; RT, reaction time. ^**^p < 0.01 for the Bonferroni-corrected pairwise comparison between LCF and NEU.

For Go Reaction Time (RT), a significant main effect of Image Type was observed [*F*(2, 44) = 6.349, *p* < 0.01, *η^2^_P_* = 0.224]. *Post-hoc* comparisons revealed that reaction times for neutral images were significantly longer than those for low-calorie food images (491.180 ± 9.421 ms vs. 470.172 ± 7.102 ms). The main effect of Group was not significant [*F*(1, 22) = 2.108, *p* = 0.161, *η^2^_P_* = 0.087]. Furthermore, no significant interaction effect was found between Group and Image Type [*F*(2, 44) = 2.522, *p* = 0.092, *η^2^_P_* = 0.103], as shown in Figure 6.

#### 3.3.2 Go Accuracy

**Figure 7:**
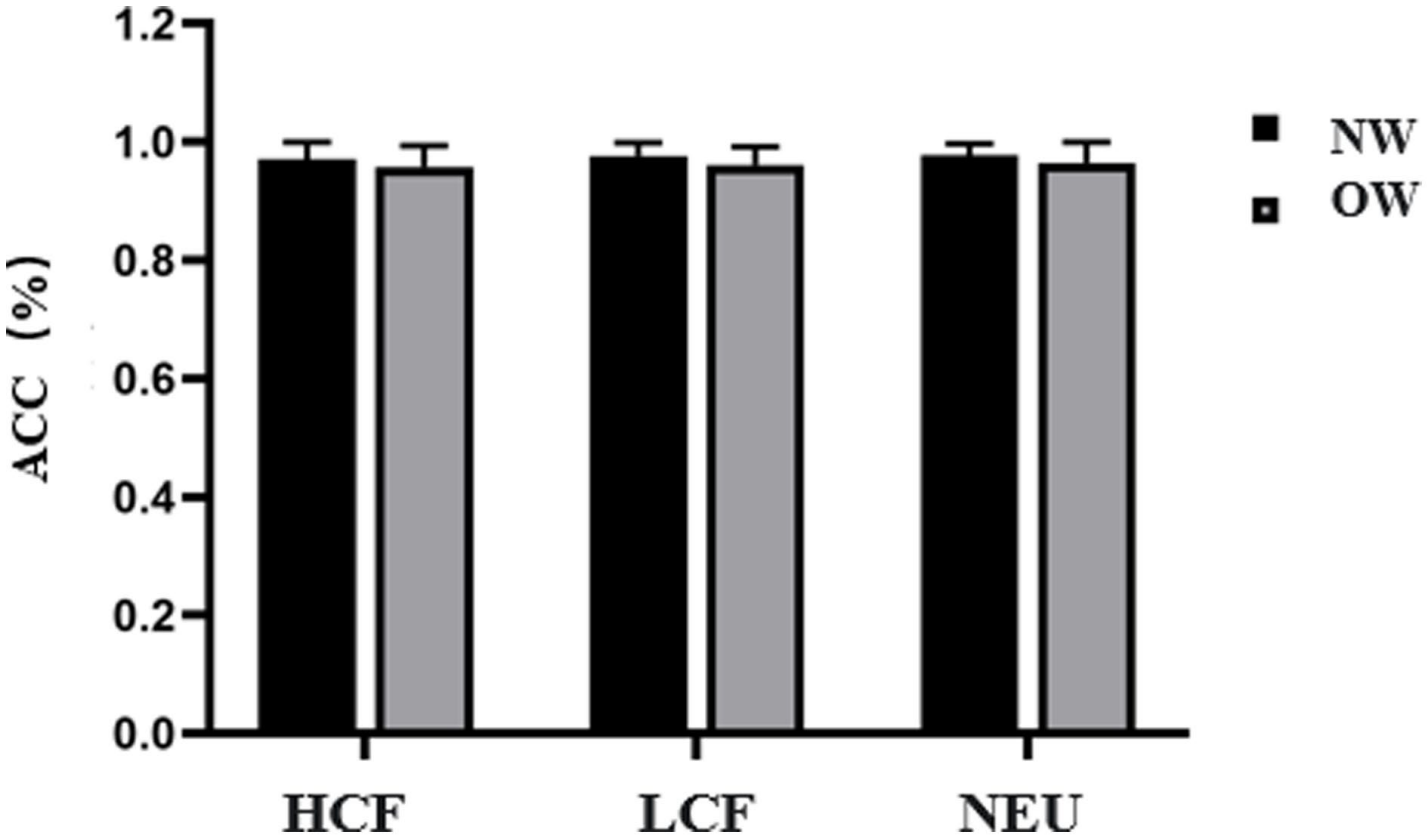
Go Accuracy for the Food Go/Nogo task in normal weight and overweight university students. Task conditions comprised high-calorie food (HCF), low-calorie food (LCF), and neutral images (NEU). Data are expressed as mean ± SD. NW, normal-weight group; OW, overweight group; ACC, accuracy.

Regarding Go Accuracy, no significant main effects were found for Group or Image Type, and the interaction effect between Group and Image Type was also not significant (*p* > 0.05), as shown in Figure 7.

#### 3.3.3 Nogo Accuracy

**Figure 8:**
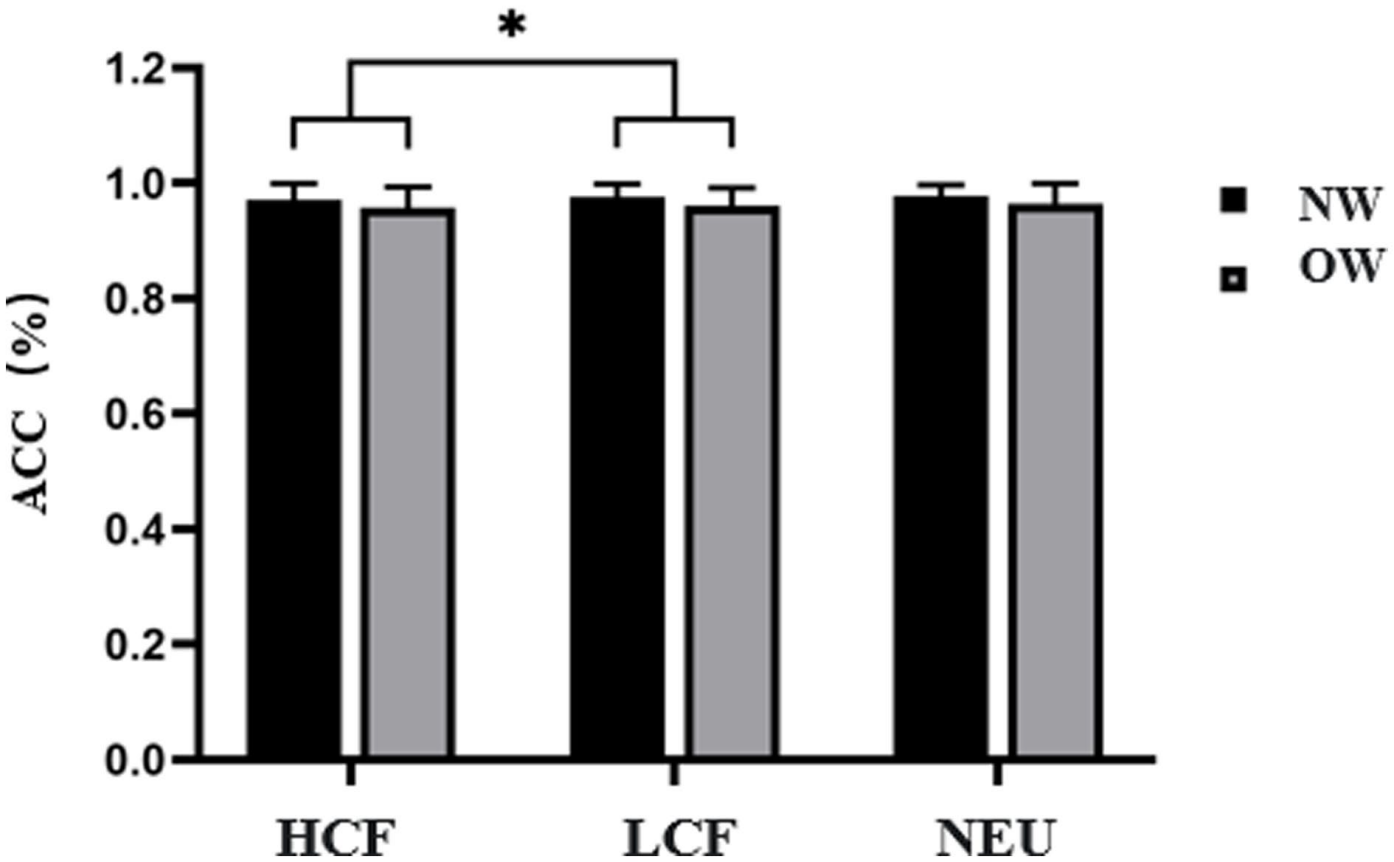
Nogo Accuracy for the Food Go/Nogo task in normal weight and overweight university students. Task conditions comprised high-calorie food (HCF), low-calorie food (LCF), and neutral images (NEU). Data are expressed as mean ± SD. NW, normal-weight group; OW, overweight group; ACC, accuracy. ^*^p < 0.05 for the Bonferroni-corrected pairwise comparison between HCF and LCF (as indicated by brackets).

For Nogo Accuracy, results indicated a significant main effect of Image Type [*F*(2, 48) = 6.133, *p* = 0.011, *η^2^_P_* = 0.204]. Specifically, accuracy for high-calorie food images was significantly higher than that for low-calorie food images (0.922 ± 0.015 vs. 0.860 ± 0.023). Neither the main effect of Group nor the interaction effect between Group and Image Type was significant (*p* > 0.05), as shown in Figure 8.

## 4 Discussion

### 4.1 Analysis of behavioral characteristics of general inhibitory function in normal-weight and overweight university students

Utilizing the Flanker task, this study investigated domain-general interference control in overweight university students. We found no significant group differences in reaction time or accuracy, indicating that general inhibitory function remains behaviorally intact in this population.

This finding diverges from previous studies focusing on clinically obese populations (Nederkoorn et al., 2006; Gunstad et al., 2007), which have consistently documented significant deficits in inhibitory control. This study proposes that the core reason for this discrepancy lies in the fundamental difference in the severity of pathophysiological burden between being overweight and being obese. Existing neurobiological evidence suggests that systemic low-grade inflammation induced by adipose tissue (e.g., elevated levels of IL-6 and CRP) is a key mechanism impairing the structure and function of the prefrontal cortex (Stillman et al., 2020). However, such impairment may have a “cumulative threshold.” In the overweight sample included in this study (BMI 24–28 kg/m ^2^), the degree of adiposity had not yet reached clinical obesity standards. Consequently, the induced metabolic disturbances and neuroinflammation levels were likely relatively mild and had not breached the critical threshold required to disrupt the prefrontal executive control network. Thus, the brain remained capable of maintaining efficient behavioral output through normal neural resource allocation.

Furthermore, the developmental characteristics and inherent neural plasticity of our sample provide a critical perspective for interpreting these null findings. Our participants were in early adulthood—specifically university students—a developmental period characterized by the zenith of physiological function and neurocognitive flexibility (Stern, 2009). Even if the overweight status exerted subtle deleterious effects on the nervous system, the robust compensatory mechanisms—often associated with the high cognitive reserve typical of educated youth—may have been sufficient to buffer against such potential interference (Sowell et al., 2001). Consequently, this resulted in “intact” performance across macroscopic behavioral indices, including reaction time and accuracy. This suggests that for young adults in the overweight stage, domain-general cognitive control capabilities remain functionally preserved. Importantly, this finding challenges the propensity in some previous studies to homogenize overweight and obese individuals as a single cognitively impaired group, thereby underscoring the imperative to rigorously distinguish between the distinct pathological stages of “overweight” and “obesity” when delineating the weight–cognition relationship (Yang et al., 2018).

Furthermore, our analyses confirmed the presence of a typical “conflict effect,” a prerequisite for interpreting inhibitory performance. Specifically, participants across both groups displayed significantly slower responses and lower accuracy rates during incongruent trials compared to congruent trials. This finding serves to validate the experimental paradigm employed, indicating that the conflicting stimuli effectively mobilized attentional resources and imposed the expected conflict monitoring demands (Eriksen and Eriksen, 1974). Notably, the interference cost—manifested as the decrement in processing efficiency under high-conflict conditions—was statistically indistinguishable between the two groups. This observation offers further corroboration that, within this cohort of university students, being overweight has not exerted a generalized deleterious impact on the fundamental ability to resolve cognitive conflict.

### 4.2 Analysis of behavioral characteristics of food-specific inhibitory function in normal-weight and overweight university students

To rigorously examine whether overweight individuals possess domain-specific deficits in inhibiting food-related impulses, this study deployed a modified Food Go/Nogo paradigm. The results revealed that the overweight group did not exhibit diminished food-specific inhibitory capacity relative to the normal-weight group. Rather, both cohorts displayed strikingly similar behavioral patterns: a generalized processing bias toward food stimuli that was independent of current weight status.

Specifically, regarding the Go trials—which index response execution and implicit approach motivation—participants in both groups demonstrated significantly accelerated reaction times for low-calorie food images compared to neutral stimuli. This observation aligns robustly with the “Incentive Salience” theory (Finlayson et al., 2007), which posits that food cues, acting as biologically primary rewards with profound evolutionary significance, automatically trigger bottom-up attentional capture (Nummenmaa et al., 2011), thereby facilitating rapid motor preparation and execution. Of note, the absence of a disproportionately faster approach bias toward high-calorie foods in the overweight group offers a nuanced insight. It implies that, within this specific university population, overweight individuals have not yet transitioned into a state of pathological “reward hypersensitivity.” Instead, their attentional and motor responsiveness to high-energy food cues remains within a homeostatic range, distinguishing them from clinical obesity phenotypes often characterized by compulsive approach tendencies.

In contrast to the automatic approach tendencies observed in Go trials, the critical Nogo trials—which index the capacity for active response inhibition—revealed a counter-intuitive yet illuminating pattern. Specifically, participants across both groups demonstrated significantly higher accuracy when inhibiting responses to high-calorie foods compared to low-calorie alternatives. While this finding seemingly contradicts the conventional “obesity–impulsivity” hypothesis, which posits that excess weight compromises the ability to resist palatability, it can be robustly interpreted through the framework of the “Goal Conflict Model of Eating” (Stroebe et al., 2017).

Within this cohort of university students, who are characterized by higher educational attainment and cognitive reserve, highly salient high-calorie cues likely served not merely as temptations (Papies, 2016), but as “conflict signals.” These signals effectively activated latent long-term goals regarding health or weight management, thereby triggering a heightened top-down recruitment of cognitive control resources to override the immediate impulse. We conceptualize this phenomenon as “defensive inhibition.” It suggests that for these overweight young adults, the “cold” executive control network (mediated by the prefrontal cortex) remains functionally intact (Heatherton and Wagner, 2011) and capable of mounting a successful counter-regulatory response even when confronted with potent dietary challenges, further distinguishing them from clinical populations with established inhibitory failure. However, because the present study did not include a clinical obesity group, this stage-based comparison remains indirect; whether “defensive inhibition” is sustained, attenuated, or shifts toward a more global inhibitory deficit with progression to clinical obesity requires designs spanning the full weight continuum.

### 4.3 Strengths, limitations, and future direction

A primary strength of this study lies in its specific focus on the “overweight” population—a critical prodromal stage in obesity progression. By moving beyond the traditional binary contrast between “clinical obesity” and “normal weight,” this research addresses a scarcity of data regarding cognitive characteristics during the early phase of weight gain. Additionally, the adoption of a task-dissociation strategy, combining the Flanker task with a modified Food Go/Nogo task, allowed for a systematic differentiation between domain-general interference control and food-specific response inhibition, providing a more nuanced understanding of the cognitive control profiles in this specific population.

However, interpretations of these findings must consider several limitations. First, the reliance on BMI as the sole anthropometric metric may have introduced classification bias; given the active university cohort, an elevated BMI could reflect increased lean body mass rather than visceral fat accumulation, potentially masking group differences associated with adiposity. Second, the cross-sectional design captures only a snapshot of the relationship between weight status and inhibition, precluding causal inferences. Moreover, the two-group comparison (overweight vs. normal weight) does not allow us to determine how the proposed “defensive inhibition” pattern may evolve with further weight gain, nor whether it eventually gives way to the domain-general inhibitory deficits more consistently reported in clinical obesity. Third, unmeasured variables, such as “restrained eating” tendencies or current hunger states, might have moderated performance in the food-specific tasks. In addition, although individuals with clinically diagnosed eating disorders were not included, more fine-grained eating-disorder features (e.g., subclinical symptomatology) and sleep patterns (e.g., sleep duration/quality prior to testing) were not formally quantified, which may have introduced modest state-dependent variability in task performance. Finally, the recruitment of university students implies a sample with relatively high cognitive reserve, which may have buffered against mild impairments, limiting the generalizability of the findings to populations with lower educational attainment.

In light of these insights, future research should incorporate multi-dimensional body composition metrics (e.g., DEXA or BIA) to verify the “adiposity-inflammation threshold” hypothesis. In addition, incorporating a clinical obesity group (i.e., a three-group design: normal weight vs. overweight vs. obesity) and, ideally, longitudinal follow-up across weight trajectories would enable a direct test of whether defensive inhibition reflects an early compensatory phase that diminishes beyond an adiposity-related threshold. Furthermore, integrating neuroimaging techniques like Event-Related Potentials (ERP) is essential to detect subtle neural compensatory mechanisms that may not be evident in behavioral data alone. Subsequent studies should also examine how psychological traits, such as restrained eating, interact with weight status, thereby facilitating the development of more precise intervention strategies.

## 5 Conclusion

The present study demonstrates that overweight university students exhibit preserved domain-general and food-specific inhibitory control, challenging the prevailing view that excess weight inevitably compromises cognitive function. The heightened accuracy observed in inhibiting high-calorie food cues points to a “defensive inhibition” mechanism, likely underpinned by the cognitive reserve inherent in this educated cohort. These findings support a dose-dependent model of neurocognitive impact, effectively differentiating the prodromal “overweight” stage from clinical obesity. Consequently, this research highlights a critical window of preserved cognition, offering a robust theoretical basis for early lifestyle interventions aimed at halting the progression of obesity.

## Data Availability

The original contributions presented in the study are included in the article/Supplementary material, further inquiries can be directed to the corresponding author.
